# Correlative Effects on Nanoplastic Aggregation in Model Extracellular Biofilm Substances Investigated with Fluorescence Correlation Spectroscopy

**DOI:** 10.3390/polym16152170

**Published:** 2024-07-30

**Authors:** Tobias Guckeisen, Rozalia Orghici, Silke Rathgeber

**Affiliations:** Institute for Integrated Natural Sciences, Physics Department, University of Koblenz, Universitätsstraße 1, 56070 Koblenz, Germany; guckeisen@uni-koblenz.de (T.G.);

**Keywords:** FCS, nanoplastics, nanoparticles, biofilm, EPS, aggregation

## Abstract

Recent studies show that biofilm substances in contact with nanoplastics play an important role in the aggregation and sedimentation of nanoplastics. Consequences of these processes are changes in biofilm formation and stability and changes in the transport and fate of pollutants in the environment. Having a deeper understanding of the nanoplastics–biofilm interaction would help to evaluate the risks posed by uncontrolled nanoplastic pollution. These interactions are impacted by environmental changes due to climate change, such as, e.g., the acidification of surface waters. We apply fluorescence correlation spectroscopy (FCS) to investigate the pH-dependent aggregation tendency of non-functionalized polystyrene (PS) nanoparticles (NPs) due to intermolecular forces with model extracellular biofilm substances. Our biofilm model consists of bovine serum albumin (BSA), which serves as a representative for globular proteins, and the polysaccharide alginate, which is a main component in many biofilms, in solutions containing Na^+^ with an ionic strength being realistic for fresh-water conditions. Biomolecule concentrations ranging from 0.5 g/L up to at maximum 21 g/L are considered. We use non-functionalized PS NPs as representative for mostly negatively charged nanoplastics. BSA promotes NP aggregation through adsorption onto the NPs and BSA-mediated bridging. In BSA–alginate mixtures, the alginate hampers this interaction, most likely due to alginate–BSA complex formation. In most BSA–alginate mixtures as in alginate alone, NP aggregation is predominantly driven by weaker, pH-independent depletion forces. The stabilizing effect of alginate is only weakened at high BSA contents, when the electrostatic BSA–BSA attraction is not sufficiently screened by the alginate. This study clearly shows that it is crucial to consider correlative effects between multiple biofilm components to better understand the NP aggregation in the presence of complex biofilm substances. Single-component biofilm model systems based on comparing the total organic carbon (TOC) content of the extracellular biofilm substances, as usually considered, would have led to a misjudgment of the stability towards aggregation.

## 1. Introduction

Biofilms consist of microbial communities that encapsulate themselves in a matrix of extracellular polymeric substances (EPS). The highly hydrated matrix in a biofilm mainly consists of polysaccharides and proteins and additionally, nucleic acids, lipids, and other biopolymers [1]. The matrix provides protection and enables colonization of microorganisms in adverse conditions. It represents a dominant fraction of the reduced carbon in soils, sediments, and suspended aggregates in ocean and freshwater ecosystems [1]. On the one hand, EPS play an important role in the environment as a nutrient for the microbiome and is therefore important for the microbial ecology; on the other hand, the microbes produce the EPS and therefore determine their composition.

Plastic pollution, as a result of the worldwide utilization of plastic products across almost all sectors, is a serious global problem. In particular, nanoplastics that originates mostly from the breakdown of microplastics is of tremendous concern, because it is believed to have higher toxicity compared to microplastics [2]. This has fueled an increasing interest in nanoplastics research [2,3].

Interactions between nanoplastics and natural organic matter (NOM), including biofilm substances, are known to have a strong impact on nanoplastic aggregation as summarized in the following review articles [4,5,6,7,8]. NOM can facilitate nanoparticle (NP) aggregation by bridging from one particle to another and by adsorbing to the NP and thereby changing its surface properties, e.g., its charge or steric hindrance.

Studying the interactions between nanoplastics and NOM is relevant for a better understanding of its environmental impact. The transport of nanoplastics in the environment can be immensely influenced if the NPs aggregate in the presence of NOM [4]. When they accumulate in biofilms, i.e., if they aggregate in the presence of biofilm substances in particular, nanoplastics can be harmful due to their inherent properties that can cause, e.g., oxidative stress and reduced growth rate [9], but also due to interactive effects between the NPs and environmental components, as they can act as vectors for contaminants and transport pollutants such as heavy metals and persistent organic pollutants into the biofilms [4,9].

The biofilm composition, i.e., the proportion of different biofilm substances, is variable and it is yet unclear how this impacts interactions between nanoplastics and biofilm substances. It is known that changing environmental conditions impacts the composition of biofilms and their EPS. The effects of climate change are altering environmental conditions on a global scale, and ecosystem disruption may exacerbate this trend. As an example, different subspecies of *Pseudomonas aeruginosa* can overproduce different components to adapt to varying conditions [10] and it has been seen that biofilm composition is dependent on nutrient availability, temperature and other environmental stress factors, such as the acidification of surface waters due to ${\mathrm{CO}}_{2}$ emissions, sulfur dioxide, or chemical waste pollution [11]. Therefore, it is important to understand how changes in biofilm composition translates into changes in interactions and aggregation with NPs to be able to assess the possible consequences.

Previous investigations on NP aggregation in the presence of NOM or exudated biomolecules [12,13,14,15,16,17,18,19,20,21,22,23,24,25,26,27,28] were conducted via dynamic light scattering (DLS) at low biomolecule concentrations below 0.2 g/L, whereas biofilm density can reach up to 100 g/L [29].

In the current study, we apply fluorescence correlation spectroscopy (FCS) [30,31] to study the aggregation of fluorescently labeled NPs in the presence of biofilm model substances. In contrast to DLS, only the fluorescently labeled species, here, NPs, contribute to the detected signal, enabling measurements on higher contents of biopolymer components and mixtures thereof, ranging here from 0.5 g/L up to 21 g/L. In FCS, a small sample volume is excited, and the fluorescence originating from this volume is detected. The movement of the species through the detection volume is causing fluctuations in fluorescence intensity. A correlation analysis of the intensity fluctuations provides information about the underlying dynamics and concentration of the labeled species. From the time dependency of the correlated signal, information about aggregation can be obtained, since the time spent in the focal volume increases with particle size. The amplitude of the signal is a direct measure of the number of moving, labeled entities, which decreases upon aggregation. Therefore, FCS has the advantage of enabling measurements at low NP concentrations without compromises in signal strength, since the signal originates from fluctuations in intensity which are amplified at low concentrations.

Both monodisperse [12,13,14,15,16,18,19,20,21,24,26,27,28] and degraded polystyrene (PS) NPs [17,22,23] were used as model system for nanoplastics. Some engineered particles were functionalized—mainly with amino and carboxylic acid groups—for electrostatic stabilization and to mimic specific interactions of nanoplastics with NOM. The main pathways to nanoplastics in the environments include mechanical degradation with no change in polymer chemistry as well as photo-thermal oxidation and hydrolysis leading to carboxylic groups [19]. Since polymers containing nitrogen are rare, negatively charged NPs with no modification or carboxylic acid functionalization are given a higher relevance than those carrying amino groups. Therefore, we use in our study non-functionalized PS NPs as representative for mostly negatively charged nanoplastics.

NPs were dispersed in natural waters [15,19] or deionized water with different ionic composition [13,14,17,18,22,26,27,28], ionic strength [13,14,17,20,21,22,26,27,28], and pH values [12,13,18,28] simulating fresh [12,14,16,18,19,24,26] and sea water conditions [13,15,17,20,21,22,27,28] with a clear focus on the latter. In model solutions the focus was put on the divalent salts ${\mathrm{CaCl}}_{2}$ and ${\mathrm{MgCl}}_{2}$ [13,14,18,19,20,26,27,28] and to some extent on the monovalent salt NaCl [13,14,21,26,27,28], being most relevant in the natural environment. NP stability was characterized in the presence of NOM from natural water or in the presence of models representing biomass decay products, i.e., refractory humic substances with a focus on the water-soluble components humic and fulvic acid, HA and FA, respectively [12,14,17,18,19,20,21,23]. Less attention has been paid to exudated biomacromolecules, like extracellular substances extracted from biofilms [15,16,22,24] or EPS analogs [12,13,16,26,27,28], like bovine serum albumin (BSA) and alginate, as a representative protein and polysaccharide, respectively. In our study, we therefore focus on model extracellular biofilm substances consisting of a protein and polysaccharide as the main components of EPS. In contrast to refs. [13,26,27,28], we focus on solutions with an ionic strength of 10 mmol/L, which is realistic for fresh water instead of sea water conditions, i.e., solutions of low ionic strength are considered. Ref. [16] did not specify the ionic strength. We chose BSA as a model protein, since it has representative properties for globular proteins and is negatively charged at pH = 7 as the majority of proteins in EPS [32] and alginate as a model polysaccharide. Alginate is a component that can be found in many biofilms and is a good model for the predominantly negatively charged polysaccharides found in EPS [33].

Interactions between NP and NOM are complex in nature and include electrostatic and van der Waals interactions, hydrogen bonding, steric repulsion, ligand formation, hydrophobic–hydrophilic interactions [25,26,27,28,34], and entropy-driven interactions due to conformational changes upon adsorption [35,36]. The interplay between the different interactions depends strongly on the pH value, salinity, and type and valence of ions present in solutions. At low ionic strength, electrostatic interactions predominate [12,13,14,16,17,18,19,20,24,26]. At high ionic strength relevant for sea water conditions, interactions other than repulsive electrostatic interactions, as stated above, become more relevant [13,15,17,20,21,22,27,28].

Despite the heterogeneity of the investigated systems, some general conclusions can be drawn about the mechanism driving and suppressing aggregation of negatively charged NPs in the presence of NOM. The prevailing evidence is that at all salinities, the presence of divalent ions supports NP aggregation due to ion-mediated bridging without [13,18,19] and with participation of NOM [13,17,18,26,27,28], i.e., due to prior adsorption of NOM onto the NPs [13,17,18,19,20,21,24,26,27,28]. The particular strong destabilization impact of alginate in the presence of ${\mathrm{Ca}}^{2+}$ was attributed to the ion-induced network (gel) formation [13]. In the presence of monovalent and divalent ions, adsorption of NOM onto the NPs can further promote NP destabilization at low NOM contents due to charge neutralization before sterical and electrostatic stabilization sets in at higher NOM contents, leading, i.e., to the formation of smaller aggregates and/or to a slowing down of the aggregation kinetics [13,14,15,16,17,19,20,21,22,24,26,27,28,37]. In the presence of ${\mathrm{Na}}^{+}$, suppression of aggregation was also observed [13,14,17,20,21,22,24,26,27,28]. There are only a few reports on stable NOM-NP solutions and negligible impact of NOM onto NP aggregation in the presence of ${\mathrm{Na}}^{+}$, ${\mathrm{Ca}}^{2+}$, and ${\mathrm{Mg}}^{2+}$ at low salinities, i.e., when electrostatic interactions dominate [14,18]. NOM adsorbs on bare and carboxyl-functionalized NPs, most likely driven by attractive interactions like hydrophobic interactions, hydrogen bonding, and/or ligand formation, predominant in particular at high salinities [13,15,17,20,21,24,26,27,28]. BSA carries hydrophobic sites as well as positive and negative patches. Strong BSA adsorption presumably also originates from structural rearrangements to a more compact globular shape upon adsorption on the NPs leading to a conformation with higher entropy [13,26,27,28]. The hydrophilic, negatively charged alginate exhibits the lowest adsorption tendency [13], but can coadsorb, e.g., with BSA on NPs, to form a micellar shell around the NPs [24].

Nevertheless, most of the previous experimental studies did not consider model mixtures and thus were limited in their interpretations by the compositional complexity of the investigated NP-NOM systems [12,13,14,15,17,18,19,20,21,22,26,27,28]. We therefore follow a different approach by reducing the problem to a simple model system comprising representatives for the main components of EPS. In addition, we limit ourselves for this first study to the monovalent ion ${\mathrm{Na}}^{+}$. Together with a systematic variation in the BSA–alginate ratio, we are in this way able to distinguish not only between the individual contributions of the protein and polysaccharide to the NP aggregation/stabilization but also to identify correlative effects due to the presence of both components. This leads to a better understanding of how changes in composition translate into changes in the interactions between nanoplastics and extracellular biofilm components and how this in turn affects nanoplastic aggregation. Stress factors, i.e., the acidification of surface waters due to ${\mathrm{CO}}_{2}$ emissions, can alter biofilm composition and impact interactions between nanoplastics and NOM. The pH value is therefore varied since it might be a result of anthropogenic environmental changes with consequences for the nanoplastics–polymer interactions due to changes in the charge nanoplastics carries in solution. Furthermore, changing the pH also allows for a systematic variation in the electrostatic interactions, giving insight into the role they play in NP aggregation.

Our studies show that in addition to electrostatic interactions, which are important at all concentrations, depletion forces are crucial at higher concentrations. Alginate drives NP aggregation by pH-independent depletion interactions. BSA promotes NP aggregation by adsorption onto the NPs and attractive BSA-BSA interactions, such as hydrogen bonding, hydrophobic interactions, and electrostatic interactions resulting from its heterogenous charge distribution, which are in competition with repulsive electrostatic interactions originating from its pH-dependent overall negative net charge at higher pH values. In protein–polysaccharide mixtures, most likely, BSA–alginate complexation leads to a screening of the attractive interactions and consequently, a stabilization of the NP solutions compared to solutions containing BSA only. Our study reveals that considering the total organic carbon (TOC) content of the extracellular biofilm substances can lead to a misestimation of NP stability in complex biofilms, since correlations between the different interactions among proteins, polysaccharides, and NP have to be considered. A simple model based on the polysaccharide concentration could be feasible, if the protein concentration is not too high.

## 2. Materials and Methods

### 2.1. Material Characterization and Sample Preparation

Reference measurements for FCS were performed on a daily basis by using carboxylate-modified PS particles. Particles (Fluospheres) were purchased from Molecular Probes, Inc. (Eugene, OR, USA). They had a diameter of 110 nm as cross-checked by DLS (Zetasizer, Malvern Panalytical, Malvern/UK) using the Stokes–Einstein equation (see Equation (5)), exhibited an absorption maximum at 505 nm and an emission maximum at 515 nm. The stock solution according to the manufacturer had a concentration of 2 wt% in water ($3.6\times 10^{13}$ particles/mL = 60 nM). For the reference measurements, a concentration of 500 pM was used corresponding to about 2 particles per detection volume. Green fluorescent non-functionalized (plain) and carboxylate-modified PS NPs were purchased from RuixiBiotechCo. Ltd. (Xi’an, China, article no. R-PGX25) labeled with 4-Chloro-7-nitrobenzofurazan (NBD-Cl) with an excitation and emission maximum of 467 nm and 539 nm, respectively. Here the concentration of the stock solution was 1 wt% (30 nM) in water, and the concentration in all FCS measurements was set to 500 pM. For the FCS measurements, the non-functionalized (plain) NPs were used as representatives for mostly negatively charged nanoplastics. The carboxylate-modified PS NPs of RuixiBiotechCo. Ltd. were measured as a control with DLS together with the Fluospheres and the non-functionalized NPs. The carboxylate-stabilized particles showed good agreement between the DLS and FCS measurements, and the results for the Fluospheres also corresponded well with the supplier’s specifications for the diameter (109 nm). The DLS and FCS measurements of the non-functionalized particles differed in size. We attributed this to an increased polydispersity compared to the carboxylate-modified particles as indicated by the higher polydispersity index determined from the DLS measurements. FCS and DLS determine the number-averaged and z-averaged sizes, respectively, where the latter puts much stronger weight on large particles.

According to the measured zeta potential, all particles were negatively charged under neutral conditions, where the deprotonated carboxyl group contributed to a higher negative charge for the functionalized particles compared to the plain ones. The NP properties are summarized in Table 1.

BSA was purchased from Biowest (Bradenton, FL, USA, article no. P6154) and alginate (alginic acid sodium salt) of low viscosity (article no. B25266) from Alfa Aesar (Haverhill, MA, USA). The viscosity of alginate was specified by the manufacturer with 30–90 mPas for a 1% blank solution in water. Sodium chloride (molecular biology grade) was purchased from VWR (Radnor, PA, USA, article no. 33614.265), and the water used in all experiments was deionized by Seradest SD 4000 (Veolia Deutschland, Berlin, Germany) with a conductivity of <0.1 μS/cm. The samples with various BSA and alginate contents were prepared by mixing a filtered solution of 10 mM NaCl with appropriate amounts of BSA and alginate from stock solutions having concentrations of 100 g/L and 10 g/L, respectively. Stock solutions were freshly prepared on the day of the experiment, or at the earliest, the preceding evening, and stored in a refrigerator. The polytetrafluoroethylene (PTFE) filters (pore size 0.45 μm) were purchased from Whatman (Little Chalfont, UK). All pH values were adjusted with HCl from fisher scientific (Waltham, MA, USA) (article no. 10458790) and NaOH from Köhler GmbH (Andernach, Germany, article no. 882219541). NPs were added last, until a final concentration of 500 pM was achieved. Samples were sonicated for 15 min, and they remained stable for several hours as shown by control measurements with FCS.

### 2.2. FCS—Setup

FCS measurements were performed with a home-built setup based on the stand of an inverted Axios Observer Z1 fluorescence microscope using an argon ion laser (LGK 7872 ML, Lasos, Jena, Germany) for fluorescence excitation at 488 nm. A dichroic mirror (H488 LPXR, 247 AHF, Tübingen, Germany) separated the emitted light from the laser excitation. A water immersion objective (LD C-Apochromat 40x, NA 1.1; Zeiss AG, Oberkochen, Germany) was used to focus and collect the illumination and fluorescence light, respectively. A pinhole (d = 50 μm, Edmund Optics, Mainz, Germany) blocked out-of-focus light in the detection path. The fluorescence light was detected by two avalanche photodiodes (Count 100-B, Laser Components, Olching, Germany) after being split by a 50% blank beam splitter (CCM1-BS013/M, Thorlabs, Newton, MA, USA). The intensity correlation function was determined by cross-correlating the signal from the two photodiodes, suppressing in this way detector after pulsing, an artefact that arises from feedback in a single-photon detector [38]. For the correlation, a photon correlator (DPC 230, Becker & Hickl, Berlin, Germany) operated by the SPCM software was used. FCS curves were calculated by a multiple-τ algorithm, within a correlation time window of 10 s. The samples were measured in a circular steel sample container with cover glass at the bottom that could be sealed to avoid evaporation.

All FCS results are the mean of triplicate measurements of at least two independent experiments with freshly prepared samples. The acquisition time was between $t_{a}$ = 150–900 s, depending on the size of the aggregates and the time required for obtaining acceptable statistics. All measurements were performed at a room temperature of 21 °C in an air-conditioned room.

### 2.3. FCS—Analysis

Assuming a Gaussian shape of the detection volume and three-dimensional Brownian diffusion, the following model function can be used to fit autocorrelation functions obtained by FCS for solutions containing *m* populations of diffusing particles with different brightness $Q_{i}$, number fraction $X_{i}$, and diffusion times $\tau_{Di}$ (*i* = 1,…, *m*) [30,31,39]:

(1) $$\begin{array}{cc} G(\tau ) & =\frac{\sum_{i=1}^{m}Q_{i}^{2}X_{i}g_{i}\left(\tau \right)}{N{\left(\sum_{i=1}^{m}Q_{i}X_{i}\right)}^{2}} \end{array}$$

(2) $$\begin{array}{cc} g_{i}\left(\tau \right) & ={\left(1+\frac{\tau }{\tau_{Di}}\right)}^{-1}{\left(1+\frac{\tau }{S^{2}\tau_{Di}}\right)}^{-\frac{1}{2}} \end{array}$$

Here, *N* is the total number of particles in the detection volume and *S* denotes the aspect ratio of the detection volume, i.e., the ratio of its vertical and lateral diameter measured parallel and perpendicular to the laser beam direction, respectively. For the investigations on partly aggregated samples, as conducted here, a model considering two diffusing populations (*m* = 2) was applied. These two populations comprised individual NPs or small aggregates (*i* = 1, diameter $d_{1}$, volume $V_{1}$), respectively, and a fraction of large aggregates (*i* = *a*, diameter $d_{a}$, volume $V_{a}$). Assuming that the brightness of an aggregate scales with the number of comprised individual NPs, Equation (1) can be rewritten with $Q_{1}$/$Q_{a}$ = $V_{1}$/$V_{a}$ = $d_{1}^{3}$/$d_{a}^{3}$ as follows:

(3) $$G\left(\tau \right)=\frac{1-X_{a}}{N\left(1-X_{a}+X_{a}{\left(\frac{d_{a}}{d_{1}}\right)}^{3}\right)}g_{1}\left(\tau \right)+\frac{X_{a}{\left(\frac{d_{a}}{d_{1}}\right)}^{6}}{N\left(1-X_{a}+X_{a}{\left(\frac{d_{a}}{d_{1}}\right)}^{3}\right)}g_{a}\left(\tau \right)$$

Here, $X_{a}$ is the number fraction of large aggregates in the detection volume. $g_{a}$ and $g_{1}$ result from Equation (2) with the diffusion times $\tau_{Da}$ and $\tau_{D1}$ of the two populations, respectively. The diffusion time measures the residence time of the particle in the detection volume. When the size of the labeled species is not negligible compared to the size of the detection volume, which is the case here when measuring aggregates of NPs, the following expression for calculating the diffusion time should be used [31,40]:

(4) $$\tau_{Di}=\frac{{\omega_{0}}^{2}+{\left(\frac{d_{i}}{2}\right)}^{2}}{4D_{i}},i=1,a$$

where the $D_{i}$’s are the diffusion coefficients of the two populations (*i* = 1, *a*), respectively, and $\omega_{0}$ is the lateral radius of the detection volume. It should also be noted that Equation (4) is only valid for $d_{i}\leq 2\omega_{0}$. The diffusion coefficient can be approximated by the Stokes–Einstein relation strictly valid for spherical particles only:

(5) $$D_{i}=\frac{k_{B}\cdot T}{3\pi \eta d_{i}},i=1,a$$

Here, $k_{B}$ is the Boltzmann constant, *T* the absolute temperature, and η the solution viscosity. The aspect ratio *S* and the lateral radius $\omega_{0}$ of the detection volume can be determined from reference measurements using spherical particles with known diameter/diffusion coefficient. Reference measurements were performed on a daily basis using carboxylate-modified Fluospheres with known diameter (see sample preparation). The diameter of the detection volume was typically $\omega_{0}$ = 350 nm, and the aspect ratio was around 11.

Even though the aggregates cannot be assumed to have a spherical shape, Equations (4) and (5) can be taken to calculate the effective hydrodynamic diameter of the aggregates. In Equation (3) polydispersity is not considered, nevertheless, it can also be used in more polydisperse systems such as aggregative systems, yielding an average value of the hydrodynamic diameter [31]. However, absolute values obtained for aggregate dimensions exceeding $2\omega_{0}\approx 700$ nm should be taken with precaution. These approximations do not represent any significant restriction for determining the onset of aggregation. The share of aggregates is presented in terms of the volume fraction $\varphi_{a}$ instead of the number fraction $X_{a}$ as it is more sensitive to the onset of aggregation.

The FCS curves were fitted using Equation (3). If the $\chi^{2}$ value of the fit without an aggregate fraction ($X_{a}=0$) was equivalent or better than the fit with aggregate fraction, then the fit with fewer parameters ($X_{a}=0$) was taken in an Occam’s razor approach. A maximum of two populations, characterized by their representative average size, were necessary for a proper description of the FCS curves.

### 2.4. Correction for Viscosity Changes

The time development of the FCS autocorrelation curve depends not only on the diameter of the particles moving through the detection volume but also on viscosity changes due to composition and concentration variations. FCS curves, e.g., will shift to larger diffusion times with increasing particle diameter but also with increasing viscosity because both result in slower diffusion (see Equations (4) and (5)). Therefore, the viscosities of the alginate–BSA solutions were measured using an MCR 502 rheometer (Anton Paar, Graz, Austria) to correct this effect. Measurements were carried out on solutions not containing NPs, since at the NP concentrations considered here, no effect of the NPs on the viscosity of the solutions could be observed. The same applied for the globular BSA. Therefore, only the concentration-dependent influence of alginate on the viscosity needed to be corrected. Results can be found in a previous publication [41] together with an example for the viscosity change corrections.

## 3. Results and Discussion

**Figure 1:**
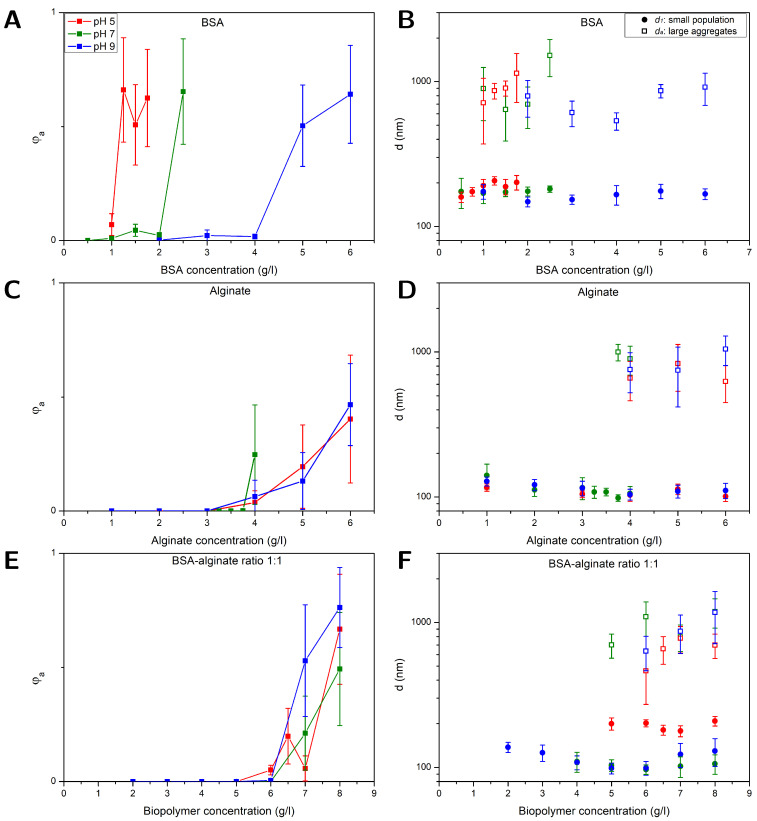
FCS results obtained at different pH values (buffer: 10 mM NaCl) for PS NPs (⌀ 100 nm) in mixtures with (**A**,**B**) BSA, (**C**,**D**) alginate, and (**E**,**F**) BSA and alginate with a fixed BSA–alginate weight ratio of 1:1. Left (**A**,**C**,**E**): volume fraction of large aggregates $\varphi_{a}$. Right (**B**,**D**,**F**): diameters of the aggregated $d_{a}$ and smaller-sized fraction $d_{1}$.

**Figure 2:**
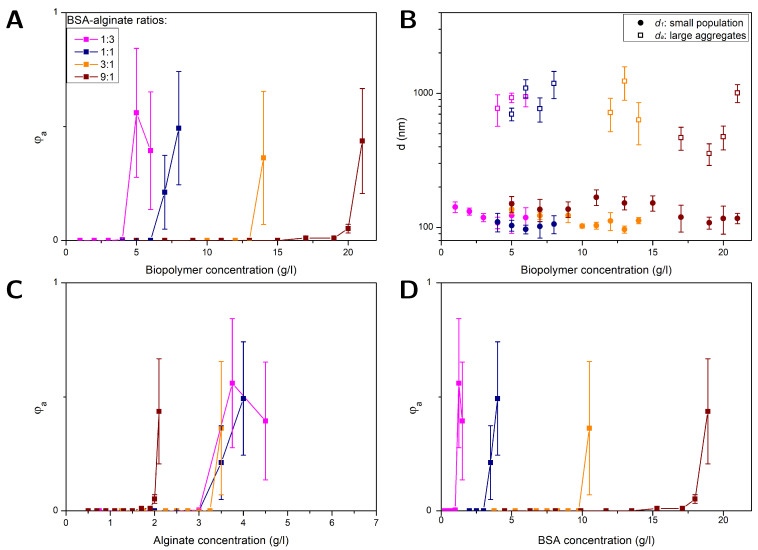
FCS results obtained for PS NPs (⌀ 100 nm) in solutions with different BSA–alginate weight ratios at pH = 7: volume fraction of aggregates $\varphi_{a}$ versus total biopolymer (BSA + alginate) (**A**), alginate (**C**), and BSA (**D**) content and the diameters of the large aggregates $d_{a}$ and smaller-sized fraction $d_{1}$ versus total biopolymer content (**B**).

The aggregation of NPs in BSA, alginate, and BSA–alginate mixtures with a fixed weight ratio of 1:1 were investigated with FCS at different pH values of 5, 7, and 9, respectively. Furthermore, at pH = 7, the BSA–alginate weight ratio (1:3, 1:1, 3:1, and 9:1) was changed. The correlation curves of the recorded fluorescence signal were fitted using Equations (3)–(5) taking into account viscosity changes due to variations in the alginate contents. The resulting volume fractions of aggregates $\varphi_{a}$ as well as the hydrodynamic diameters of the smaller-sized population $d_{1}$ and large aggregates $d_{a}$ are plotted in Figure 1 and Figure 2 as a function of the BSA, alginate, and total biopolymer content, respectively, for biopolymer concentrations ranging from 0.5 g/L up to a maximum of 21 g/L. Error bars represent the standard error of the mean obtained from evaluating triplicate measurements of two independent experiments on fresh samples.

### 3.1. NP Aggregation in BSA Solutions

Figure 1A shows that the fraction of aggregates $\varphi_{a}$ sharply increased at a certain BSA concentration. As pH decreased, the onset of aggregation decreased. The diameters of the two populations, $d_{1}$ and $d_{a}$, are shown in Figure 1B. BSA has a high affinity to the hydrophobic surfaces of the PS NPs through its hydrophobic units and covered the NPs to some extent. Independent of the BSA concentration, the average diameter of the smaller population was 175 ± 17 nm, which corresponds to a layer thickness of around 38 ± 12 nm if compared to the diameters of the plain particles. The driving force for adsorption of the first protein layer at non-functionalized hydrophobic interfaces is dominated by hydrophobic interactions [42] and BSA adsorbs readily on a PS surface [26,27,28,43,44,45]. Co-adsorption of positive ions into the NP-BSA contact zone can inhibit the evolution of high electrostatic potentials [26,27,28,46,47]. In addition, BSA adsorption is probably driven by structural changes resulting in a higher conformational entropy in the adsorbed state for the protein [13,26,27,48]. In multiple layers, protein–protein interactions are more important; those can be electrostatic interactions due to the heterogeneous charge distribution of positive and negative patches on the BSA [26,27,28,49,50], but hydrogen bonding and hydrophobic interactions could also be relevant. BSA dimensions are described as an ellipsoid with the dimensions of 14×4×4 nm [44] and BSA preferably adsorbs in a side orientation on non-functionalized PS surfaces [44].

A layer thickness of 38 ± 12 nm would correspond to about 7–13 layers of BSA, which would be considerably more layers than the previously reported mono- or bilayers of BSA on PS surfaces. More likely, small aggregates of two or three particles that could not be resolved as an additional species were causing the increase in size. The ability of FCS to resolve small aggregates is limited, especially if there is only a small fraction of aggregates present, e.g., a fraction of 10% can only be resolved under very good signal per particle conditions if the sizes differ at least by a factor of about 2.6 [51]. If the fraction or the signal per particle is lower, here, 6 kHz per particle compared to 15 kHz per particle in a more ideal scenario, the size difference has to be even larger. The aggregation of the BSA-covered PS NPs was most likely caused by BSA-mediated bridging [52]. Despite the negative charge of the PS NPs (see Table 1) and BSA, protein adsorption together with BSA-BSA bridging due to attractive NP–protein and protein–protein interactions, as described above, can induce aggregation. The enhanced aggregation of the NPs at low pH values can be explained by a decreasing net charge of the BSA and consequently, reduced electrostatic repulsion between the BSA-covered NPs. The isoelectric point of BSA is at pH 5 [53], i.e., at this point, the BSA molecules carry no net charge, and attractive interactions are dominant [27,49,50]. A lack of electrostatic repulsion between BSA molecules obviously promotes BSA-mediated bridging of the NPs which is in accordance with the early onset of NP aggregation at pH = 5. At higher pH values, the onset of aggregation is shifted to higher BSA concentrations. This is in line with the increasing absolute value of the net BSA surface potential that changes from 0 mV at pH 5 over −22 mV at pH 7 to −29 mV at pH 9 [54], leading to enhanced electrostatic stabilization of the BSA-covered NPs with increasing pH. Within the error bars, no significant dependence of the aggregates size on the pH value and BSA content could be observed.

### 3.2. NP Aggregation in Alginate Solutions

Figure 1C,D display results obtained for $\varphi_{a}$, $d_{a}$ and $d_{1}$ from measurements on alginate–NP mixtures at different pH values as a function of the alginate concentration. In comparison to the BSA-NP mixtures, aggregation set in between 3 and 4 g/L, independent of the pH value. Alginate carries negatively charged carboxylic acid sides. Unlike BSA, the hydrophilic, negatively charged alginate should not possess significant affinity to the hydrophobic, negatively charged PS particles. Therefore, the NPs should not be covered with alginate, which is consistent with the observation that the diameters obtained for the smaller sized fraction $d_{1}$ = 112 ± 14 nm (see Figure 1D) equaled the diameters of the pristine NPs (97 ± 7 nm) within the errors. The driving force for the aggregation arises from a depletion effect, which occurs between large particles in a solution with polymeric substances like alginate. The alginate, which is smaller compared to the NPs, is excluded from gaps between NPs when they approach distances smaller than the alginate size, i.e., on the order of twice the alginate radius of gyration. As a result, the osmotic pressure between polymer (alginate) solution and depletion zone between the NPs leads to an attractive net force pushing the particles together [55,56,57]. This effect has already been observed for PS particles in both sodium PS sulphonate and alginate solutions [57,58]. Indicated by the absence of a clear pH effect, electrostatic interactions did not appear to play a major role in alginate–NP mixtures.

### 3.3. NP Aggregation in BSA-Alginate Mixtures

Figure 1E,F depict results for $\varphi_{a}$, $d_{a}$ and $d_{1}$ from studies on NPs in BSA–alginate mixtures with a weight ratio of 1:1 plotted as a function of the overall biopolymer content, i.e., BSA plus alginate. The results are similar to those obtained for NPs in alginate solutions. An increase in the volume fraction of aggregates at alginate concentrations similar to those observed for NP–alginate solutions and no evidence of a pH effect on aggregation were observed. NPs in BSA solutions aggregated at lower concentrations (between 1 and 2 g/L) than solutions containing BSA–alginate 1:1 mixtures. Thus, although BSA accounted for half of the biopolymer content, there was no indication that attractive BSA-BSA interactions, which were impacted by the pH-dependent net electrostatic charge of the protein in the solutions containing BSA only, drove aggregation. This rather suggests that primarily depletion interactions drove aggregation as in the alginate–NP solutions.

Bridging of NPs by BSA in pristine BSA solutions is based on attractive, electrostatic interactions between positive and negative patches of the protein [49,50]. The negatively charged carboxylic acid sides on alginate can bind to the positively charged protein sides to form BSA–alginate complexes, thus hampering the protein–protein attractive interactions [59]. This also agrees with previous studies of other authors that have shown that alginate and other polyelectrolytes can stabilize protein-covered particles [60]. The fact that alginate hinders protein–protein interactions and prevents NP bridging by BSA may indicate that alginate is not completely excluded from the surface, as assumed in classical depletion, but that the entropic or enthalpic penalty for the presence of alginate at the particle surface is insufficient and that there is a finite concentration of alginate, termed weak depletion [61]. However, compared to the NP–alginate mixtures, the increase in $\varphi_{a}$ in the NP–BSA–alginate mixtures was somewhat steeper and could be caused by BSA-mediated bridging once the alginate was (partly) depleted from the NP contact zones.

The diameters of the smaller-sized population and aggregated fraction in alginate–BSA 1:1 mixtures are shown in Figure 1F. The diameters of the smaller-sized population $d_{1}$ = 194 ± 14 nm at pH 5 were considerably larger than those obtained at pH 7 and 9 ($d_{1}$ = 112 ± 14 nm). The $d_{1}$ values obtained at pH = 5 resembled more the dimension of the species observed in BSA solutions, i.e., most likely small aggregates (see Figure 1B), while values obtained at pH = 7 and 9 were similar to the ones obtained for the single NPs in alginate solutions (see Figure 1D). Alginate complexation with free or/and adsorbed BSA presumably hampered the attractive protein–protein interactions at all pH values investigated [24,59]. At pH = 7 and pH = 9 at which BSA carries a negative net charge, this might be sufficient to prevent multiple-layer formation on the NPs and BSA-BSA bridging. At pH = 5, BSA does not carry a net charge, reducing repulsive interactions between the BSA and enabling that attractive interactions remain to some extent. An intricate interplay of attractive and repulsive interactions determines whether BSA is adsorbed in multiple layers, bridging between particles occurs, or protein aggregates are formed, e.g., many proteins need a critical nucleus for aggregation, so an additional barrier for the formation of this nucleus has to be overcome [62]. Therefore, the formation of multiple BSA layers and BSA-BSA bridging can occur without simultaneous aggregation of BSA, which, however, cannot be excluded on the basis of the current results.

Figure 2A,B depict results obtained for $\varphi_{a}$, $d_{a}$ and $d_{1}$ at pH = 7 for different protein–polysaccharide weight ratios (1:3, 1:1, 3:1 and 9:1) as a function of the total biopolymer concentration, i.e., alginate plus BSA. Up to a BSA–alginate weight ratio of (3:1) the diameters $d_{1}$ = 114 ± 14 nm obtained for the smaller-sized fraction (see Figure 2B) were comparable to the results obtained for alginate–NP solutions. The values approximately corresponded to the dimensions of single NPs. There were no indications for multiple-BSA-layer adsorption nor BSA-BSA bridging. In the BSA–alginate (9:1) mixture, BSA-BSA interactions were most likely not sufficiently screened by the alginate, leading to slightly increased dimensions of about $d_{1}$ = 136 ± 21 nm.

The biofilm substances and EPS are often measured without differentiation between proteins and polysaccharides as TOC content. The combined biopolymer concentration in Figure 2A,B reflects what is encountered when only TOC is considered. Figure 2C,D compare the results obtained for $\varphi_{a}$ shown in Figure 2A as a function of the biopolymer content, now plotted versus alginate and BSA concentration, respectively. The concentration at which aggregation started increased with a higher protein portion since the alginate was hampering the BSA-BSA interactions. BSA was then contributing only to the biopolymer concentration but did not cause aggregation.

While aggregation always started at different BSA concentrations (see Figure 2D), aggregation started for BSA–alginate weight ratios of 1:3, 1:1 and 3:1 at the same alginate concentration, the value being comparable to that obtained for NPs in pure alginate solutions. In these mixtures, the alginate was mainly causing the aggregation by depletion interactions. Thus, a model that only considers the alginate concentration could be useful as long as the protein–polysaccharide ratio is not too high. At high BSA contents, attractive BSA-BSA interactions were not efficiently screened by the alginate, shifting the aggregation threshold to lower alginate concentrations. However, protein–polysaccharide ratios of 9:1 and more actually occur and demand more complex models. A simple model based solely on alginate or BSA would lead to a misjudgment of the aggregation behavior, i.e., an overestimation or underestimation of the NP stability, respectively. These results emphasize the importance of knowing the composition of biofilm substances rather than just measuring the TOC content.

## 4. Conclusions

FCS was used to investigate the aggregation of nanoplastics in model extracellular biofilm substances consisting of the partly positively charged and partly hydrophobic protein BSA and the anionic polysaccharide alginate, and mixtures thereof, at selected pH values. It was shown that both biofilm components influenced the aggregation of negatively charged, hydrophobic PS NPs in a significant and specific way, and that correlative effects originating from the presence of both components could not be neglected. Alginate drove NP aggregation by pH-independent depletion interactions. Attractive BSA-BSA interactions, which are known to be due to electrostatic interactions between positive and negative patches of the protein, hydrogen bonding, and hydrophobic interactions, were responsible for the aggregation of BSA-covered PS particles in pure BSA solutions. Repulsive electrostatic BSA-BSA interactions due to the pH-dependent negative net charge of BSA reduced the aggregation tendency of the NPs at pH values above 5, i.e., the isoelectric point of BSA. In the presence of alginate, these interactions were (partly) screened, and depletion forces became more relevant for NP aggregation, which would be in accordance with the ability of BSA to form complexes with alginate. For a certain range of BSA–alginate ratios, the aggregation of the NPs was mainly driven by depletion interactions. However, at high BSA contents, the attractive BSA-BSA interaction was not sufficiently screened, leading to an aggregation behavior not sufficiently reproduced by a single-component model, neither pure alginate nor BSA. Furthermore, the present work demonstrated that a model for biofilm substances consisting of a single component, which is based on comparing the TOC content of extracellular biofilm substances, a path often followed for the sake of simplicity, would have led to an underestimation of the stability towards aggregation. However, for a certain range of protein–polysaccharide ratios (here up to 3:1), a simple model based on comparing the polysaccharide contents instead of the total biopolymer concentration might be feasible to predict NP aggregation under environmental conditions. Therefore, it is of utmost importance to know the composition of biofilm substances including the protein and polysaccharide fraction to better understand the NP stability in the presence of complex biofilm substances, particularly if results obtained for different systems are compared.

## Figures and Tables

**Table 1:** Nanoparticle (NP) properties obtained from dynamic light scattering (DLS) and fluorescence correlation spectroscopy (FCS).

	Fluospheres (COOH)	R-PGCX25 (COOH)	R-PGX25 (Plain)
Diameter supplier	109 nm	100 nm	100 nm
Hydrodynamic diameter (FCS)	reference ^1^	105 ± 6 nm	97 ± 7 nm
Hydrodynamic diameter (DLS)	111 ± 2 nm	105 ± 2 nm	124 ± 2 nm
Polydispersity Index (DLS)	0.038	0.031	0.073
Zeta potential (DLS)	−52 ± 4 mV	−51 ± 8 mV	−28 ± 4 mV

^1^ The Fluosphere measurements are used to calibrate the FCS setup.

## Data Availability

Data are available on request.

## Abbreviations

The following abbreviations are used in this manuscript:

FCS	Fluorescence correlation spectroscopy
PS	Polystyrene
NP(s)	Nanoparticle(s)
BSA	Bovine serum albumin
EPS	Extracellular polymeric substances
NOM	Natural organic matter
DLS	Dynamic light scattering
HA	Humic acid
FA	Fulvic acid
TOC	Total organic carbon

## References

[1] Flemming H.C., Wingender J. (2010). The biofilm matrix. Nat. Rev. Microbiol..

[2] Wang J., Zhao X., Wu F., Niu L., Tang Z., Liang W., Zhao T., Fang M., Wang H., Wang, X (2021). Characterization, occurrence, environmental behaviors, and risks of nanoplastics in the aquatic environment: Current status and future perspectives. Fundam. Res..

[3] Deschênes L., Ells T. (2020). Bacteria-nanoparticle interactions in the context of nanofouling. Adv. Colloid Interface Sci..

[4] Alimi O.S., Budarz J.F., Hernandez L.M., Tufenkji N. (2018). Microplastics and Nanoplastics in Aquatic Environments: Aggregation, Deposition, and Enhanced Contaminant Transport. Environ. Sci. Technol..

[5] Ge Z., Lu X. (2023). Impacts of extracellular polymeric substances on the behaviors of micro/nanoplastics in the water environment. Environ. Poll..

[6] Pradel A., Catrouillet C., Gigault J. (2023). The environmental fate of nanoplastics: What we know and what we need to know about aggregation. NanoImpact.

[7] Cid-Samamed A., Diniz M.S. (2023). Recent Advances in the Aggregation Behavior of Nanoplastics in Aquatic Systems. Int. J. Mol. Sci..

[8] Corsi I., Bergami E., Grassi G. (2020). Behavior and Bio-Interactions of Anthropogenic Particles in Marine Environment for a More Realistic Ecological Risk Assessment. Front. Environ. Sci..

[9] Shi X., Chen Z., Wei W., Chen J., Ni B.-J. (2023). Toxicity of micro/nanoplastics in the environment: Roles of plastisphere and eco-corona. Soil Environ. Health.

[10] Mann E.E., Wozniak D.J.L. (2012). Pseudomonas biofilm matrix composition and niche biology. FEMS Microbiol. Rev..

[11] Moryl M., Kaleta A., Strzelecki K., Rozalska S., Rozalski A. (2014). Effect of nutrient and stress factors on polysaccharides synthesis in Proteus mirabilis biofilm. Acta Biochim. Pol..

[12] Oriekhova O., Stoll S. (2018). Heteroaggregation of nanoplastic particles in the presence of inorganic colloids and natural organic matter. Environ. Sci. Nano.

[13] Liu Y., Huang Z., Zhou J., Tang J., Yang C., Chen C., Huang W., Dang Z. (2020). Influence of environmental and biological macromolecules on aggregation kinetics of nanoplastics in aquatic systems. Water Res..

[14] Cai L., Hu L., Shi H., Ye J., Zhang Y., Kim H. (2018). Effects of inorganic ions and natural organic matter on the aggregation of nanoplastics. Chemosphere.

[15] Grassi G., Gabellieri E., Cioni P., Paccagnini E., Faleri C., Lupetti P., Corsi I., Morelli E. (2020). Interplay between extracellular polymeric substances (EPS) from a marine diatom and model nanoplastic through eco-corona formation. Sci. Total Environ..

[16] Barros C.H.N., Fulaz S., Vitale S., Casey E., Quinn L. (2020). Interactions between functionalised silica nanoparticles and Pseudomonas fluorescens biofilm matrix: A focus on the protein corona. PLoS ONE.

[17] Yu S., Shen M., Li S., Fu Y., Zhang D., Liu H., Liu J. (2019). Aggregation kinetics of different surface-modified polystyrene nanoparticles in monovalent and divalent electrolytes. Environ. Pollut..

[18] Zhang F., Wang Z., Wang S., Fang H., Wang D. (2019). Aquatic behavior and toxicity of polystyrene nanoplastic particles with different functional groups: Complex roles of pH, dissolved organic carbon and divalent cations. Chemosphere.

[19] Song Z., Yang X., Chen F., Zhao F., Zhao Y., Ruan L., Wang Y., Yang Y. (2019). Fate and transport of nanoplastics in complex natural aquifer media: Effect of particle size and surface functionalization. Sci. Total Environ..

[20] Tallec K., Blard O., Gonzales-Fernandez C., Brotons G., Berchel M., Soudant P., Huvet A., Paul-Pont I. (2019). Surface functionalization determines behavior of nanoplastic solutions in model aquatic environments. Chemosphere.

[21] Wu J., Jiang R., Lin W., Ouyang G. (2019). Effect of salinity and humic acid on the aggregation and toxicity of polystyrene nanoplastics with different functional groups and charges. Environ. Pollut..

[22] Mao Y., Li H., Huangfu X., Liu Y., He Q. (2020). Nanoplastics display strong stability in aqueous environments: Insights from aggregation behaviour and theoretical calculations. Environ. Pollut..

[23] Xu Y., Ou Q., He Q., Wu Z., Ma J., Huangfu X. (2021). Influence of dissolved black carbon on the aggregation and deposition of polystyrene nanoplastics: Comparison with dissolved humic acid. Water Res..

[24] Liu Y., Yue T., Liu L., Zhang B., Feng H., Li S. (2023). Molecular assembly of extracellular polymeric substances regulating aggregation of differently charged nanoplastics and subsequent interactions with bacterial membrane. J. Hazard. Mater..

[25] Grillo R., Rosa A.H., Fraceto L.F. (2015). Engineered nanoparticles and organic matter: A review of the state-of-the-art. Chemosphere.

[26] Li X., He E., Xia B., Liu Y., Zhang P., Cao X., Zhao L., Xu X., Qiu H. (2021). Protein corona-induced aggregation of differently sized nanoplastics: Impacts of protein type and concentration. Environ. Sci. Nano.

[27] Li X., He E., Jiang K., Peijnenburg W., Qiu H. (2021). The crucial role of a protein corona in determining the aggregation kinetics and colloidal stability of polystyrene nanoplastics. Water Res..

[28] Huang Z., Chen C., Liu Y., Liu S., Zeng D., Yang C., Huang W., Dang Z. (2022). Influence of protein configuration on aggregation kinetics of nanoplastics in aquatic environment. Water Res..

[29] Bishop P.L., Zhang T.C., Fu Y. (1995). Effects of biofilm structure, microbial distributions and mass transport on biodegradation processes. Water Sci. Technol..

[30] Ries J., Schwille P. (2012). Fluorescence correlation spectroscopy. Bioessays.

[31] Koynov K., Butt H.J. (2012). Fluorescence correlation spectroscopy in colloid and interface science. Curr. Opin. Colloid Interface Sci..

[32] Laspidou C.S., Rittmann B.E. (2002). A unified theory for extracellular polymeric substances, soluble microbial products, and active and inert biomass. Water Res..

[33] Subramanian S.B., Yan S., Tyagi R.D., Surampalli R.Y. (2010). Extracellular polymeric substances (EPS) producing bacterial strains of municipal wastewater sludge: Isolation, molecular identification, EPS characterization and performance for sludge settling and dewatering. Water Res..

[34] Hu B., Liu R., Liu Q., Lin Z., Shi Y., Li J., Wang L., Li L., Xiao X., Wu Y. (2022). Engineering surface patterns on nanoparticles: New insights into nano-bio interactions. J. Mater. Chem. B.

[35] Kopac T. (2021). A Protein corona, understanding the nanoparticle–protein interactions and future perspectives: A critical review. Int. J. Biol. Macromol..

[36] Saptarshi S., Duschl A., Lopata A.L. (2013). Interaction of nanoparticles with proteins: Relation to bio-reactivity of the nanoparticle. J. Nanobiotechnol..

[37] Okshevsky M., Gautier E., Farner J.M., Schreiber L., Tufenkji N. (2020). Biofilm formation by marine bacteria is impacted by concentration and surface functionalization of polystyrene nanoparticles in a species-specific manner. Environ. Microbiol. Rep..

[38] Zhao M., Jin L., Chen B., Ding Y., Ma H., Chen D. (2003). Afterpulsing and its correction in fluorescence correlation spectroscopy experiments. Appl. Opt..

[39] PicoQuant, Practical Manual for Fluorescence Microscopy Techniques, Fluorescence Correlation Spectroscopy (FCS). https://www.picoquant.com/images/uploads/page/files/17319/5_fcs.pdf.

[40] Starchev K., Zhang J., Buffle J. (1998). Applications of Fluorescence Correlation Spectroscopy—Particle Size Effect. J. Colloid Interface Sci..

[41] Guckeisen T., Orghici R., Rathgeber S. (2023). Probing the tendency for aggregation of nanoplastics in model extracellular biofilm substances with fluorescence correlation spectroscopy. Single Mol. Spectrosc. Superresolution Imaging XVI Proc. Spie.

[42] Yoon J.Y., Kim J.H., Kim W.S. (1999). The relationship of interaction forces in the protein adsorption onto polymeric microspheres. Colloids Surf. A Physicochem. Eng. Asp..

[43] Zsom R.L.J. (1986). Dependence of preferential bovine serum albumin oligomer adsorption on the surface properties of monodisperse polystyrene latices. J. Colloid Interface Sci..

[44] Wangkam T., Yodmongkol S., Disrattakit J., Sutapun B., Amarit R., Somboonkaew A., Srikhirin T. (2012). Adsorption of bovine serum albumin (BSA) on polystyrene (PS) and its acid copolymer. Curr. Appl. Phys..

[45] Rabe M., Verdes D., Seeger S. (2011). Understanding protein adsorption phenomena at solid surfaces. Adv. Colloid Interface Sci..

[46] Dulm P.V., Norde W., Lyklema J. (1981). Ion participation in protein adsorption at solid surfaces. J. Colloid Interface Sci..

[47] Elgersma A.V., Zsom R.L.J., Norde W., Lyklema J. (1990). The adsorption of bovine serum albumin on positively and negatively charged polystyrene latices. J. Colloid Interface Sci..

[48] Norde W., Anusiem A.C.I. (1992). Adsorption, desorption and re-adsorption of proteins on solid surfaces. Coll. Surf..

[49] Grant M.L. (2001). Nonuniform Charge Effects in Protein-Protein Interactions. J. Phys. Chem. B.

[50] Kubiak-Ossowska K., Jachimska B., Mulheran P. (2016). How Negatively Charged Proteins Adsorb to Negatively Charged Surfaces: A Molecular Dynamics Study of BSA Adsorption on Silica. J. Phys. Chem. B.

[51] Meseth U., Wohland T., Rigler R., Vogelc H. (1999). Resolution of Fluorescence Correlation Measurements. Biophys. J..

[52] Chen K., Xu Y., Rana S., Miranda O.R., Dubin P.L., Rotello V.M., Sun L., Guo X. (2011). Electrostatic Selectivity in Protein-Nanoparticle Interactions. Biomacromolecules.

[53] Guckeisen T., Hosseinpour S., Peukert W. (2019). Isoelectric Points of Proteins at the Air/Liquid Interface and in Solution. Langmuir.

[54] Salis A., Boström M., Medda L., Cugia F., Barse B., Parsons D.F., Ninham B.W., Monduzzi M. (2011). Measurements and Theoretical Interpretation of Points of Zero Charge/Potential of BSA Protein. Langmuir.

[55] Mao Y., Cates M.E., Lekkerkerker H.N.W. (1995). Depletion force in colloidal systems. Physica A.

[56] Smith N.J., Williams P.A. (1995). Depletion Flocculation of Polystyrene Latices by Water-soluble Polymers. J. Chem. Soc. Faraday Trans..

[57] Sharma A., Tan S.N., Walz J.Y. (1997). Effect of Nonadsorbing Polyelectrolytes on Colloidal Interactions in Aqueous Mixtures. J. Colloid Interface Sci..

[58] Bondy C. (1939). The creaming of rubber latex. Trans. Faraday Soc..

[59] Zhao Y., Carvajal M.T., Harris M.T. (2009). Interactions between bovine serum albumin and alginate: An evaluation of alginate as protein carrier. J. Coll. Int. Sci..

[60] Sabet S., Seal C.K., Swedlund P.J., McGillivray D.J. (2020). Depositing alginate on the surface of bilayer emulsions. Food Hydrocoll..

[61] Garcia A.G., Nagelkerke M., Tuinier R., Vis M. (2020). Polymer-mediated colloidal stability: On the transition between adsorption and depletion. Adv. Colloid Interface Sci..

[62] Auer S., Dobson M.D., Vendruscolo M. (2007). Characterization of the nucleation barriers for protein aggregation and amyloid formation. HFSP J..

